# Effects of increasing the PSA cutoff to perform additional biomarker tests before prostate biopsy

**DOI:** 10.1186/s12894-017-0281-8

**Published:** 2017-10-03

**Authors:** Tobias Nordström, Jan Adolfsson, Henrik Grönberg, Martin Eklund

**Affiliations:** 1 Department of Medical Epidemiology and Biostatistics, Karolinska Institutet, S-171 77 Stockholm, Sweden; 2 Department of Clinical Sciences at Danderyd Hospital, Karolinska Institutet, S-182 88 Stockholm, Sweden; 3 Department of Clinical Science, Intervention and Technology, Karolinska Institutet, Stockholm, Sweden; 4 Swedish Agency for Health Technology Assessment and Assessment of Social Services, Stockholm, Sweden

**Keywords:** Prostate cancer, Prostate neoplasm, Prostate-specific antigen (PSA), Biomarker, Stockholm3, STHLM3

## Abstract

**Background:**

Multi-step testing might enhance performance of the prostate cancer diagnostic pipeline. Using PSA >1 ng/ml for first-line risk stratification and the Stockholm 3 Model (S3M) blood-test >10% risk of Gleason Score > 7 prostate cancer to inform biopsy decisions has been suggested. We aimed to determine the effects of changing the PSA cutoff to perform reflex testing with S3M and the subsequent S3M cutoff to recommend prostate biopsy while maintaining the sensitivity to detect Gleason Score ≥ 7 prostate cancer.

**Methods:**

We used data from the prospective, population-based, paired, diagnostic Stockholm 3 (STHLM3) study with participants invited by date of birth from the Swedish Population Register during 2012–2014. All participants underwent testing with PSA and S3M (a combination of plasma protein biomarkers [PSA, free PSA, intact PSA, hK2, MSMB, MIC1], genetic polymorphisms, and clinical variables [age, family, history, previous prostate biopsy, prostate exam]). Of 47,688 men in the STHLM3 main study, we used data from 3133 men with S3M >10% and prostate biopsy data. Logistic regression models were used to calculate prostate cancer detection rates and proportion saved biopsies.

**Results:**

44.2%, 62.5% and 67.9% of the participants had PSA <1, <1.5 and <1.7 ng/ml, respectively. Increasing the PSA cut-off for additional work-up from 1 ng/ml to 1.5 ng/ml would thus save 18.3% of the performed tests, 4.9% of the biopsies and 1.3% (10/765) of Gleason Grade ≥ 7 cancers would be un-detected. By lowering the S3M cutoff to recommend biopsy, sensitivity to high-grade prostate cancer can be restored, to the cost of increasing the number of performed biopsies modestly.

**Conclusion:**

The sensitivity to detect prostate cancer can be maintained when using different PSA cutoffs to perform additional testing. Biomarker cut-offs have implications on number of tests and prostate biopsies performed. A PSA cutoff of 1.5 ng/ml to perform additional testing such as the S3M test might be considered.

**Trial registration:**

ISRCTN84445406.

## Background

Recently, Crawford and colleagues proposed an approach of using PSA 1.5 ng/ml as first-line testing before using biomarker-based tests to inform prostate biopsy decisions [1]. Such a multi-step work-up is an attractive approach for improving prostate cancer diagnostics. Men with PSA below the population median carries a low risk to develop metastatic or lethal disease also during long follow-up [2]. Since testing with PSA has high availability and low cost, base-line PSA testing is a attractive for efficient first-line risk stratification [2].

For second-line testing, the S3M (Stockholm3 Model) blood-test has been developed, including data on proteins, a genetic score and clinical information (age, digital rectal examination, prostate volume, and previous biopsy) [3]. Compared with both organized PSA-screening and current prostate cancer testing (without organized screening but with high rates of PSA testing), the STHLM3 studies have shown that use of the S3M test may decrease both the number of prostate biopsies and over-diagnosis, while maintaining sensitivity to high-grade disease [3, 4]. This was done using PSA >1 ng/ml as cutoff for performing the S3M blood-test and a risk of high-grade disease exceeding that of PSA = 3 ng/ml to indicate recommendation for prostate biopsies.

With the approach suggested by Crawford et al. [1], two thirds of men would be identified as having a very low risk of developing high-grade disease. Compared with a lower PSA cutoff, using 1.5 ng/ml would decrease the number of performed biomarker tests. However, while performing the biomarker test for fewer men, it would also yield a smaller pool of men in which to identify prostate cancer cases, potentially affecting overall sensitivity.

It is unknown how changing the PSA cut-off for performing a reflex test affects the overall diagnostic sensitivity, the number of performed biopsies, and number of performed biomarker tests. We therefore illustrate such effects for the first-line test PSA and the second-line biomarker test S3M.

## Methods

STHLM3 (ISRCTN84445406) is a prospective and population-based prostate cancer diagnostic study conducted 2012–2014 including men between 50 and 69 years of age [3]. The S3M test is a blood test based on a model including a combination of plasma protein biomarkers (PSA, free PSA, intact PSA, hK2, MSMB, MIC1), genetic polymorphisms (232 SNPs), and clinical variables (age, family, history, previous prostate biopsy, prostate exam). The test gives a prediction on the individual risk of finding Gleason Score ≥ 7 on prostate biopsies, where ≥10% risk was considered increased risk in the main study. The 10% risk cutoff was choosen because it represent equal sensitivity to detect Gleason Score ≥ 7 cancer as PSA = 3 ng/ml, used in major screening studies [5]. The exact cut-off used can be chosen to fit different individuals and healthcare systems [6]. As for September 2017, the S3M test is clinically availiable for analysis at Karolinska University Laboratory, Stockholm, Sweden.

Of 47,688 participants in the STHLM3 study, 26,458 men had a PSA ≥ 1 ng/ml and underwent further testing with S3M. By design, a prostate biopsy was recommended to men with ≥10% risk of high-grade prostate cancer as predicted by PSA (≥3 ng/ml) or the S3M test. Gleason Score ≥ 7 (ISUP ≥2) defined high-grade cancer. 65.0% of participants with high risk followed the recommendation to undergo prostate biopsy during the main study period. For this analysis we included 3133 men in the STHLM3 validation cohort with biopsy data and an S3M test ≥10%.

We calculated detection rates and proportion saved biopsies when S3M was used as a reflex test after a range of a priori choosen PSA cutoff levels, keeping overall sensitivity fixed at the same level as PSA ≥ 3 (or, equivalently, S3M ≥ 10% as a reflex test in men with PSA ≥ 1). Data on men with less than 10% risk of Gleason Score ≥ 7 prostate cancer was thus incomplete. To calculate results for this group of men, we imputed case status of each non-biopsied man using Bernoulli experiments with the risk prediction from the S3M as parameter [7].

## Results

44.2%, 62.5% and 67.9% of the participants in the population-based STHLM3 study had PSA <1, <1.5 and <1.7 ng/ml, respectively. Solely increasing the cut-off for additional work-up from 1 ng/ml to 1.5 ng/ml would thus save 18.3% of the performed tests, 4.8% of the biopsies and only 1.3% (10/765) of Gleason Grade ≥ 7 cancers would be un-detected (Table 1). Participant characteristics in men with PSA ≥ 3 or S3M ≥ 10% risk of Gleason Score ≥ 7 cancer and thus undergoing a prostate biopsy are described in Table 2.

**Table 1:** Prevalence of prostate cancer different PSA ranges for men with S3 M ≥ 10% risk of Gleason Score ≥ 7 cancer. Number of men with respective finding among 47,688 men in the STHLM3 study of which 3133 had a S3 M test >10% and a subsequent prostate biopsy

PSA ng/ml	Proportion of men by PSA in STHLM3 [3] % (n)	Men with high risk of PCa (S3 M > 10%) % (n)	Gleason Score (GS) n (%)			
			3 + 3	3 + 4	4 + 3	≥4 + 4
0–0.9	21,230 (44.2)	0 (0)	N/A	N/A	N/A	N/A
1–1.4	8777 (18.3)	100 (3.2)	24 (3.1)	5 (1.1)	2 (2.0)	0 (0)
1.5–1.6	2593 (5.4)	54 (1.7)	17 (2.2)	3 (0.6)	0 (0.0)	0 (0)
1.7–1.9	2993 (6.2)	105 (3.4)	35 (4.5)	10 (2.1)	0 (0.0)	1 (0.7)
2.0–2.9	5906 (12.3)	394 (12.6)	96 (12.4)	67 (14.2)	18 (11.5)	7 (5.0)
3.0–3.9	2721 (5.7)	817 (26.1)	218 (28.2)	110 (23.3)	34 (21.7)	25 (17.9)
>4.0	3808 (7.9)	1663 (53.0)	382 (49.5)	277 (58.7)	103 (65.6)	107 (76.4)
Total	47,688 (100)	3133 (100)	772 (100)	472 (100)	157 (100)	140 (100)

**Table 2:** Cohort description. Characteristics of 3133 men in the STHLM3 study [3] with S3 M test indicating ≥10% risk of prostate cancer

Variable	
Participants, n	3133
Age, years (mean, SD)	63.4, 5.0
PSA, ng/ml (median, IQR)	6.1, 2.9
S3 M test, % risk Gleson Score ≥ 7 cancer (median, IQR)	0.20, 0.17
*Biopsy findings (n, %)*	
Benign	1578, 50.4
Gleason Score 6 (ISUP 1)	772, 24.6
Gleason Score 3 + 4 (ISUP 2)	472, 15.1
Gleason Score 4 + 3 (ISUP 3)	157, 5.0
Gleason Score ≥ 4 + 4 (ISUP ≥4)	140, 1.8

**Fig. 1:**
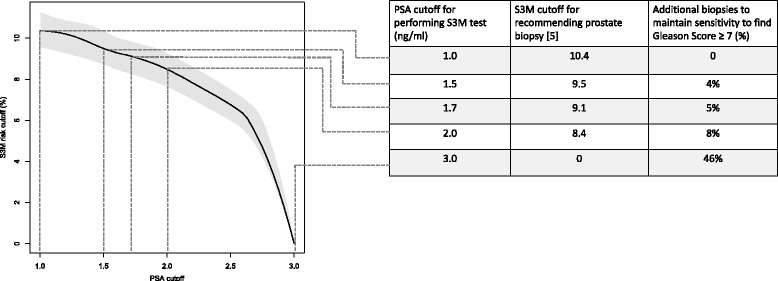
The minimum PSA used to perform the S3 M test by the minimum risk of high-grade prostate cancer as predicted by the S3 M test used to recommend prostate biopsy in order to maintain relative sensitivity compared to when using PSA = 3 ng/ml. Shaded area indicates 95% confidence interval. Data from 3133 men in the STHLM3 study with a S3 M test >10% and a subsequent prostate biopsy

To infer the mortality benefit of early detection of prostate cancer reported in ERSPC, the sensitivity to detect high-grade disease needs to be at least as high as when performing systematic prostate biopsies with a PSA cut-off of 3 ng/ml, being the threshold primarily used for biopsy in ERSPC [5]. To adjust for the slightly decreased cancer detection when increasing the first-line PSA threshold from 1 to 1.5 ng/ml, the S3M cutoff to recommend biopsy can be tuned, as previously illustrated [7]. Figure 1 illustrates how the PSA cutoff to perform the S3M test and the S3M cutoff to recommend biopsy are inter-related to maintain sensitivity to high-grade disease. For example, the total number of biopsies would increase slightly by 4% if increasing the cutoff for performing the S3M test from PSA 1.0 ng/ml to 1.5 ng/ml while maintaining sensitivity to high-grade prostate cancer (Fig. 1). A small number of Glason Score ≥ 4 + 3 were detected in low PSA ranges (Table 1). If choosing PSA 2 ng/ml for threshold to perform S3 M testing, only 1.0% (3/197) of Gleason Score ≥ 4 + 3 cancers would be undetected, but missing also 3.8% (18/472) of Gleason Score 3 + 4 cancers. Availiable number of higher-grade cases were to small in low PSA ranges for additional analyses such as in Fig. 1 on this endpoint.

## Discussion

With a possibly increasing complexity of the diagnostic chain including a multi-step approach with PSA, additional biomarker-based algorithms, and imaging before deciding to recommend further work-up, several cut-offs need to be adjusted to optimize performance. Here, we illustrate the effects of simultaneously tuning both the PSA cut-off for performing the reflex test S3M and the S3M cutoff for recommending a prostate biopsy. Using this approach, the sensitivity to detect high-grade disease can be maintained, while the number of prostate biopsies is slightly affected.

From a health-economical point of view, it is efficient to maximize the use of cheap tools such as PSA early in the diagnostic chain, with the more expensive and specialized tests used downstream. Further, as many men with low risk of high-grade disease as possible should be identified early in the process, without being subjected to additional tests or extended workup. Thus, it is interesting both from the perspective of an individual and from the healthcare system to explore how an increased cut-off to perform e.g. the S3M test might be done without compromising the overall diagnostic performance.

This analysis was based on prospective, population-based data from the STHLM3 study. It illustrates the relationship between two sequential diagnostic tests when maintaining sensitivity to detect high-grade disease. The detection rates in low PSA intervals are well comparable with previously presented data [8] and the internal validity of these data is high. While extrapolation outside the STHLM3 context is hard, corresponding analyses regarding other suggested reflex tests are warranted. Limitations of this work include lack of external validation, lack of true disease prevalence while men with <10% risk of Gleason Score ≥ 7 cancer as predicted by both PSA and S3M did not undergo prostate biopsy, and lack of long-term follow-up.

## Conclusion

We conclude that the sensitivity to detect prostate cancer can be maintained while avoiding a substantial proportion of reflex tests and biopsies by carefully choosing the PSA cutoff to perform additional testing. For instance, a PSA cutoff of 1.5 ng/ml to perform additional biomarker tests such as the S3 M test might be considered.

## Abbreviations

5-ARI: 5-alfa reductase inhibithor
ERSPC: European Randomized Study of Screening for Prostate Cancer
PSA: prostate-specific antigen
S3 M: stockholm3 model
STHLM3: Stockholm3 study
